# Conserving and Sharing Taro Genetic Resources for the Benefit of Global Taro Cultivation: A Core Contribution of the Centre for Pacific Crops and Trees

**DOI:** 10.1089/bio.2018.0017

**Published:** 2018-11-02

**Authors:** Andreas W. Ebert, Logotonu M. Waqainabete

**Affiliations:** ^1^ Freelance Consultant, Schwaebisch Gmuend, Germany.; ^2^ Pacific Community (SPC), Land Resources Division, Centre for Pacific Crops and Trees, Suva, Fiji.

**Keywords:** *Colocasia esculenta*, plant genetic resources, *in vitro* conservation, taro leaf blight, taro genetic improvement, participatory on-farm evaluation

## Abstract

This review article gives an account of the origin, domestication, and dispersal of taro, a staple food crop in many countries in the humid tropics and subtropics. Genetic diversity studies indicated that distinct gene pools exist in all the regions where taro may be naturally distributed—the Indian subcontinent, China, Southeast Asia, and in Oceania. The Asian gene pool presented the highest genetic diversity. Diploid taro is prevalent in the Pacific Islands, while both diploids and triploids are found in mainland Asia. Triploids are thought to provide better adaptability and enhanced hardiness to higher altitudes and latitudes where sexual reproduction is not viable. The Centre for Pacific Crops and Trees (CePaCT) conserves *in vitro* close to 70% of the taro genetic resources held *ex situ* and is therefore considered the world center for taro genetic resources. *Phytophthora colocasiae* or taro leaf blight (TLB) is the most severe disease of taro' causing 25%–50% yield losses and postharvest decay of corms. The CePaCT genebank supported the participatory TLB breeding program in Samoa through the provision of diverse taro germplasm from the Asian gene pool. However, CePaCT not only serves taro producers in the Pacific but also shares new allelic diversity of taro globally. More recent distributions of taro genetic diversity to West and Central Africa were in response to an outbreak and spread of TLB in West Africa. Global dissemination of taro genetic diversity is assisting producer countries in the process of adaptation to emerging biotic and abiotic stresses, exacerbated by climate change.

## Introduction

Taro (*Colocasia esculenta* (L.) Schott) is an important member of the Araceae family and a staple food crop in many countries in the humid tropics and subtropics.^1^ Before the start of the global trade and transport of agricultural commodities, taro was the world's most widely cultivated starch crop, extending from India and Southeast Asia to Northeast Asia, the Pacific Islands, Madagascar, Africa, and the Mediterranean.^2^

Global taro production reached 10.1 million tons harvested from 1.5 million hectares in 2014, resulting in an average yield of 6.945 tons per hectare.^3^ Nigeria is the largest producer with a global share of 32.4%, followed by China, Cameroon, Ghana, and Papua New Guinea (PNG). Among the top five producer countries, China, with 19.3 tons per hectare, reaches the highest productivity.^3^

While the corms and cormels are the most widely consumed plant part, the leaf blades, petioles, stolons, and inflorescences are also eaten, depending on the cultivar and local food habits.^1^ However, for efficient corm production, leaves cannot be harvested at a young age, when they are best for consumption. Taro types are well adapted to different environments ranging from swidden fields in shifting cultivation, rainfed agriculture, and home gardens to paddy fields and swamps in China^4^ and elsewhere. As is common to root and tuber crops, cultivated taro is vegetatively propagated through offshoots (suckers) from the central corms or by sets which consist of part of the leaf stalk together with a portion of the sucker corm. Wild and naturalized taros propagate through stolons,^1,5^ but flowering and seed setting are also common.^4^ The latter is hardly observed in cultivated taros.

This article reviews the origin, domestication, and dispersal of taro, its genetic diversity and *ex situ* germplasm collections, and the role of the Centre for Pacific Crops and Trees (CePaCT). CePaCT is known as the global center for taro genetic resources and mandated to introduce, conserve, and share new taro genetic diversity with farmers, plant breeders, and other scientists. A concerted effort between the CePaCT genebank, introducing new taro genetic diversity in Pacific Island countries, and plant breeders helped overcome taro leaf blight (TLB) which had devastated the taro industry in Samoa in 1993.^6^ Given the more recent outbreak and spread of TLB in West Africa (Cameroon, Ghana, and Nigeria), CePaCT distributed taro germplasm globally to assist a wide range of producer countries with new genetic diversity for better adaptation to emerging biotic and abiotic stresses.

## Origin, Domestication, and Dispersal of Taro

It is believed that taro originated in the tropics ranging from India to Indonesia.^2^ This is supported by genetic diversity studies conducted by Chaïr et al.^7^ which revealed that diversity was greater in accessions obtained from Asia compared to the Pacific, Africa, and the Americas. Within the Asian gene pool, India presented the highest numbers of alleles and private alleles.^7^

In its natural habitat taro is a semiaquatic tropical herb. It evolved into *C. esculenta* cv. *aquatilis* which is widely distributed in southern Asia, Southeast Asia, the Pacific Islands, and southwest China.^4^ Wild-type taro has relatively small corms, bears long, thin stolons, a short petiole, has green leaves, and tastes unpleasantly bitter. Through human selection, wild-type taro evolved into five main types, described as thick-petiole type with poor corm development, multi-inflorescence type, single corm type, multicormel type, and the stolon type, all of which are found in Yunnan province of China.^4^ The natural range of wild-type taro may extend up to Australia and New Guinea but is unlikely to have reached Polynesia due to sea barriers.^2^

Taro might be among the oldest cultivated crops as archaeological studies indicate that taro was already used 28,000 years ago in the Solomon Islands.^8^ Residue analyses of starch granules provide a lead to taro processing during the early and mid-Holocene in wetland areas of PNG.^9^ Dating of starch grains found in southwest Viti Levu, Fiji confirms early taro cultivation in the Pacific from 3050 to 2500 BCE.^10^

Given the higher genetic diversity of the Indian gene pool, one could argue that taro was domesticated in India and spread from there to the Asia-Pacific region with subsequent diversion of the two gene pools due to distance.^7^ Another hypothesis is that taro was domesticated independently in India and the Asia-Pacific region. According to Lebot^11^ a secondary domestication could have taken place in New Guinea. Sexual reproduction was the most likely route of taro diversification in Asia and the Pacific.^7^ All cultivars in the Pacific are diploid, and many flower and hybridize naturally due to the activity of insect pollinators^11^ resulting in a high clonal richness index.^7^ In contrast, clonal propagation combined with natural mutations appears to be a major driver of taro diversification in Africa and the Americas.

As taro is not native to Africa and the Americas, it is believed that taro reached these continents through human migration. In West Africa, most cultivars are likely of Indian origin.^7^ Taro cultivars in Madagascar can be traced to India and Indonesia, and this crop was among the first plants introduced by Austronesian settlers during the first and second Millennium CE.^12^ South African taro cultivars have a shared lineage with Japan.^7^ According to Blench,^13^ it is likely that taro was introduced to Africa together with bananas and the greater yam (*Dioscorea alata* L.). Madeira was located on the route of Portuguese traders who sourced spices in India, and taro might have been introduced to Madeira between the 14th and 15th centuries.^7^ Taro is also found in the wild or cultivated on other Macaronesian Islands (the Azores, Canary Islands, Cape Verde) that were important bases during the intensive Columbian Exchange between the 15th and 16th century.^14,15^ The Caribbean Islands received their taro cultivars from the Pacific, while Costa Rican cultivars can be traced to India or to admixtures between the Indian and Asian groups.

## Genetic Diversity of Taro

Genetic diversity studies on taro have been undertaken with tests for ribosomal DNA,^16^ chloroplast DNA,^17^ mitochondrial DNA,^16^ analysis of randomly amplified polymorphic DNA,^18^ isozymes,^17,19,20^ Amplified fragment length polymorphism markers,^21^ and microsatellite markers.^7^ These studies indicate that distinct gene pools exist in all the regions where taro may be naturally distributed—the Indian subcontinent, China and Southeast Asia (Sunda continental region), and Oceania (Sahul continental region).^2,18,19,21,22^ The Asian gene pool presented the highest genetic diversity as indicated by the number of private alleles and Shannon index.^7^

Genetic diversity studies conducted by Kreike et al.^22^ revealed that diversity among Indonesian diploid cultivars was unexpectedly high and resulted in two separate clusters, one with a clear relationship with the Pacific gene pool and the other one with the Asian gene pool. This can be explained by the fact that Irian Jaya is part of the island of New Guinea but is an Indonesian province. Indonesian provinces are located on both sides of the Wallace line which separates the Sunda plate (Southeast Asia) from the Sahul plate comprising New Guinea, Australia, and Tasmania.^23^ The majority of the diploid cultivars from Thailand also originated from the Pacific.^22^

Diploids (2n = 2x = 28 chromosomes) and triploids (2n = 3x = 42 chromosomes) are common in taro, while tetraploids are quite rare.^2^ Triploids arise when unreduced gametes (1n = 2x = 28) from one parent flower meet normal gametes (1n = 1x = 14) from another parent flower. Diploid taro is prevalent in the Pacific Islands, while triploids are found in mainland Asia.^2^ In his genetic diversity studies, Chaïr et al.^7^ demonstrated a clear grouping of diploids from the Asia-Pacific region and diploids and triploids from Indian origin. It appears that all triploids can be attributed to the Indian gene pool or might have emerged from hybridization between the Indian and Asia-Pacific gene pools and spread from there to other countries. Within the Indian gene pool, triploids are genetically quite similar to diploids, supporting the idea of a generative multiplication of the chromosome set.^7,24^

Surveys in China, India, and Nepal revealed that triploids predominate at higher altitudes and latitudes.^17,20^ Only diploids are exclusively found in China's extreme south (Hainan province), a mixture of diploids and triploids exists in southern China (Yunnan province), and only triploids are encountered in central, eastern, and northern China.^20^ In unfavorable environments for the natural breeding of diploids, the additional chromosome set likely provides triploid taros with better adaptability and the enhanced hardiness needed to survive the conditions at higher elevations and latitudes.^17,20,24^

## Taro Germplasm Collections and the Role of the CePaCT

The CePaCT of the Pacific Community (SPC), originally known as the Regional Germplasm Centre, was established in 1998 with funding from the Australian Government and the European Union. CePaCT and its genetic resources program is one of the four pillars of the Land Resources Division of SPC. CePaCT aims to assist Pacific Island Countries and Territories in the process of conservation and sustainable utilization of their genetic resources. At the same time, the Center facilitates access to improved germplasm through the acquisition of new sources of genetic diversity from other international genebanks and through the sharing of elite germplasm derived from crop improvement programs in the Pacific. Currently, CePaCT maintains the largest taro collection (1136 accessions) comprising germplasm from the Pacific and South East Asia. In addition, the Center conserves other important crops of the region, including yam (330 accessions), sweet potato (324), banana (157), swamp taro (66), and potato (54). It also has smaller collections of bele (*Abelmoschus manihot*), breadfruit, cassava, pandanus, ginger, pineapple, sugarcane, sandalwood, and vanilla. CePaCT's entire collection comprises 2151 accessions, and the Center primarily uses *in vitro* technology to conserve its crop collections at its Plant Tissue Culture Laboratory in Suva, Fiji.

Except for *in vitro* and screenhouse screening trials conducted within CePaCT, characterization and evaluation of CePaCT's taro collection are mostly done by the national programs in the recipient countries where the material is exposed to contrasting agroecological conditions. Country-level information is fed back to CePaCT and captured in its database. Up to now, the CePaCT collections were duplicated for security reasons at the tissue culture laboratory of the University of the South Pacific, Alafua Campus, Samoa. Discussions are currently in progress to establish another safety duplication site at the New Caledonian Agronomic Institute (IAC).

Research undertaken by CePaCT has focused, for example, on taro micropropagation, including the use of the bioreactor system to mass propagate elite taro cultivars for commercial planting; screening swamp taro cultivars for salt tolerance *in vitro* and in the screenhouse; cryopreservation of taro and other edible aroids; evaluation of the impact of *Dasheen mosaic virus* and other related viruses on taro yield; and participatory plant breeding to develop leaf blight tolerant taro varieties with good market traits. Efforts are currently underway to explore the use of cryopreservation for long-term conservation of major crops conserved at CePaCT to complement *in vitro* conservation.

According to the World Information and Early Warning System (WIEWS) on Plant Genetic Resources for Food and Agriculture, a total of 1685 taro (*Colocasia* spp.) accessions were held *ex situ* in 2017 by 14 institutes^25^ (Table 1). The CePaCT (FJI049) established by the Land Resources Division of the Pacific Community (SPC) is by far the largest holding institute conserving 1165 taro accessions under *in vitro* conditions. Most taro collections are maintained as field collections. Only Malaysia maintains its taro holdings both in the field and as an *in vitro* collection, and two accessions are maintained as seed (Table 1). Based on WIEWS data, CePaCT conserves close to 70% of the taro genetic resources held *ex situ* and is therefore considered the world center for taro genetic resources.

**Table 1.** Global Taro (*Colocasia* spp.) Genetic Resources Held *Ex Situ* in 2016, Based on World Information and Early Warning System

*Country*	*Holding institute code*	*No. of accessions*	*Mode of conservation*
Cuba	CUB006	112	Field
Ecuador	ECU023	18	Field
Ethiopia	ETH085	138	Field
Fiji	FJI049	1165	*In vitro*
Guyana	GUY021	8	Field
Japan	JPN183	29	Field
Malawi	MW1041	111	Field
Malaysia	MYS220	47	Field + *in vitro*
Panama	PAN172	1	*In vitro*
Peru	PER045	6	Field
South Africa	ZAF062	35	Field^a^
Spain	ESP172	3	Field
Swaziland	SWZ015	11	Field
Taiwan	TWN001	1	Seed
Total		1685	

FAO, 2018.^25^

^a^ Thirty-four accessions stored in field and one accession stored as seed in medium- and long-term storage.

Genesys, a global gateway to plant genetic resources, was developed by Bioversity International in collaboration with the Crop Trust and the Secretariat of the International Treaty on Plant Genetic Resources for Food and Agriculture (ITPGRFA) and launched in 2011 (www.genesys-pgr.org/welcome). It provides accession-level information pooled from a range of different portals such as SINGER, EURISCO, and GRIN. The Genesys portal lists only the taro genetic resources held by CePaCT in Fiji, the Centro de Conservación Agrícola de Tenerife (ESP172), Spain, and the World Vegetable Center (TWN001), Taiwan, but gives a detailed account of the countries of origin^26^ (Table 2). CePaCT's database is still under development, but will become publicly available during the second half of 2018 as an integral part of the Pacific Agricultural Information System (http://presto.thepais.net/Presto/home/home.aspx).

**Table 2.** Countries of Origin of World Taro Collection (1165 Accessions) Held by the CePaCT (FJ1049)

*Country*	*No. of accessions*	*Country*	*No. of accessions*
Indonesia	235	PNG	206
Samoa	176	Solomon Islands	99
Vanuatu	72	Vietnam	66
Philippines	60	Fiji	59
United States	41	Thailand	30
New Caledonia	24	Niue	23
Palau	18	Cook Islands	15
Malaysia	12	Japan	10
Tonga	8	French Polynesia	7
Micronesia	3	Other	1

Based on Genesys Data.^26^

PNG, Papua New Guinea; CePaCT, Centre for Pacific Crops and Trees.

It is doubtful whether the WIEWS holding list, which is built on crop/germplasm statistics provided by each country at certain intervals, is complete as it does not comprise taro holdings in India and Southeast Asia, except Malaysia. From a survey conducted by the Global Crop Diversity Trust, the significant taro collections of India^27^ (1118 accessions), PNG^27^ (700), and Indonesia^27^ (685) are not listed in the WIEWS database.

The CePaCT taro holdings comprise accessions from the Pacific Island countries, the highest number provided by PNG, Samoa, and Solomon Islands, and from several Southeast Asian countries, Indonesia being the largest contributor (Table 2). The CePaCT collection also holds accessions from Hawaii.

The CePaCT genebank conserves a regional taro core collection originating from Oceania which has been developed based on phenotypic and molecular characterization.^28^ In total, 2199 accessions of taro germplasm have been collected under the *TaroGen* (Taro Genetic Resources: Conservation and Utilization) project, funded by the Australian Agency for International Development (AusAID). Germplasm was collected from the following 10 countries in Oceania: PNG, Solomon Islands, Vanuatu, New Caledonia, Fiji, Palau, Niue, Tonga, Cook Islands, and Samoa and maintained *ex situ* at the national level. About 10% of accessions from each country were selected based on phenotypic data and taro-specific simple sequence repeat markers to contribute to a regional core collection. DNA fingerprinting data revealed great allelic diversity among accessions from PNG^29^ and the Solomon Islands.^28^

Field genebanks are commonly used for the conservation of taro genetic resources in the Pacific and other taro growing regions. However, field genebanks are always highly vulnerable due to their exposure to the risks of biotic and abiotic stresses and high maintenance costs.^30^ Taro is no exception to these threats, and significant losses in taro field collections in the Pacific have been reported by Taylor et al.^31^

Alternative conservation strategies for vegetatively propagated crops and species with recalcitrant seeds are *in vitro* culture under slow growth for short-to-medium term conservation and cryopreservation for long-term conservation. *In vitro* conservation has several advantages as accessions are not subjected to the risks of climate variability and pest and disease outbreaks, and tissue culture procedures also provide a means to eliminate most surface pathogens. Moreover, pathogen testing (virus-indexing) is routinely done to ensure that germplasm is distributed without the risk of spreading pathogens. However, *in vitro* conservation carries the risk of somaclonal variation, especially in the presence of growth regulators. At CePaCT, growth regulators are only added to the medium at the initial establishment and multiplication stage. During the maintenance phase only standard Murashige and Skoog medium is used, which is expected to have minimal effect on somaclonal variation, if any.

**FIG. 1.**
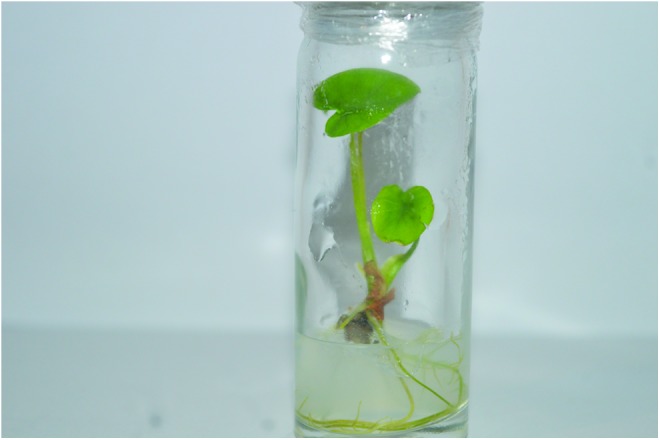
Taro plantlet in solid Murashige and Skoog (MS) medium at CePaCT. CePaCT, Centre for Pacific Crops and Trees.

**FIG. 2.**
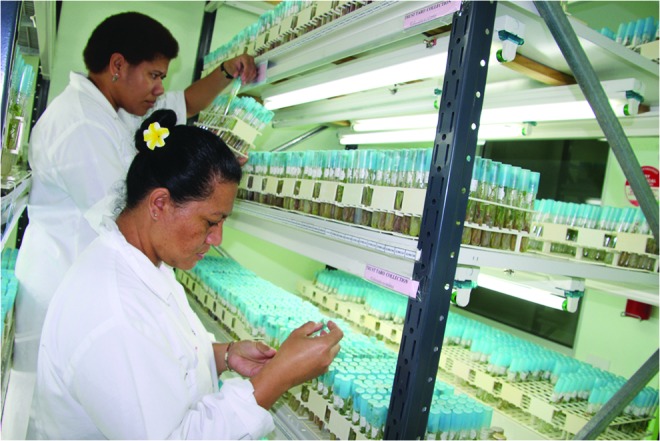
Inspection of taro cultures in the conservation room at CePaCT. Photo used with permission.

At CePaCT, subculture intervals for taro are in the range of 9–12 months, and the minimum growth conservation conditions are 23–24°C at 50%–70% relative humidity with a photoperiod of 16/8 h day/night and a photon flux density of 18.5 μEm^−2^s^−1^ provided by daylight type fluorescent lamps. Ten tubes with one plantlet each per accession are maintained for conservation purposes (Figs. 1 and 2). Subculture intervals of three years have been reported for taro when stored in the dark at a temperature of 9°C.^32^ However, the maintenance of such low-temperature storage conditions is problematic in developing countries, and not all genotypes might respond equally favorably under such extreme conditions. Conscious of the danger of somaclonal variation, CePaCT is planning to conduct experiments on extending the current subculturing period and to start screenhouse and field testing for true-to-typeness of its first taro accessions. These are dating back to 1999 when the genebank started its operation, hence are now already 19 years old.

Storage under cryopreservation at ultralow temperatures using liquid nitrogen (−196°C) is a proven alternative for taro long-term conservation. A new droplet vitrification protocol improved the post-thaw regeneration rates from the range of 21%–30% to 73%–100%.^33^ In liquid nitrogen, all cellular division and metabolic processes are suspended, thus, allowing plant material to be stored in perpetuity. Compared to field genebanks, cryopreservation offers a safe option for long-term conservation of vegetatively propagated crops and crops with intermediate or recalcitrant seeds as demonstrated for coffee.^30^ While the initial investment in cryopreservation facilities is considerable, maintenance of the cultures in cryopreservation is quite cost-effective provided that regular liquid nitrogen supply is not a problem, which is not always assured in developing countries.

Establishing national taro field genebanks and a regional core collection maintained by the CePaCT have been important steps in the conservation and sustainable use of taro genetic resources in the Pacific.^31^ These efforts should be complemented by *in situ* or on-farm conservation strategies to preserve a wider range of taro genetic resources with locally preferred traits by farmers and consumers, thereby strengthening the link between conservation and use. While CePaCT has been attested to have very efficient germplasm conservation, health testing, and distribution mechanisms in place, the Center, in general, distributes its germplasm through government agencies, and the national capacities are not always adequate to handle tissue-cultured material effectively and efficiently so that germplasm shared by CePaCT does not always reach and benefit the farmers as end users. Efforts are currently underway to engage with other national stakeholders such as NGOs, private sector companies, producer associations, churches, research organizations, and universities to establish other complementary germplasm distribution and testing pathways which might help to reach farmers more effectively.

## Fighting TLB Disease in Samoa and at Global Level Through Networking and Sharing Genetic Resources

Taro is a staple starchy food found all over the Pacific Island countries and is of great cultural and economic importance. It is the most important root crop in Samoa and was its top export earner up to 1993, when an outbreak of TLB, caused by *Phytophthora colocasiae* Racib., started to inflict severe damage to the crop and soon reached epidemic proportions due to the widespread dominance of the commercial cultivar “Niue.”^6^ However, the disease did not only affect the cultivar “Niue” but all 11 traditional cultivars grown in Samoa at that time, a clear sign of lack of genetic diversity.

TLB is the most severe disease of taro causing 25%–50% yield loss and postharvest decay of the corms.^34^ Two mating types, A1 and A2, are known, and the disease is likely to have originated on Hainan Island, China. The causal agent of TLB was first described by Raciborksi as *Phytophthora colocasiae* in 1890 from Indonesia, followed by reports on TLB in Taiwan in 1911, India in 1913, Philippines in 1925, Sri Lanka and Malaysia in 1939, Hawaii in 1941, Marinas, Carolines, and Burma in 1943, Solomon Islands in 1960, PNG in 1963, Trust Territories of Pacific Islands in 1971, Africa and the Caribbean in 1978, and American Samoa and Samoa in 1993.^6,34^

The impact of TLB on Samoa, the erosion of taro genetic resources in the Pacific and the threat of the disease to other Pacific Island countries, gave rise to the TaroGen project mentioned in the previous section. It started in 1998 and led to the collection of around 2200 taro accessions from 10 Pacific Island countries, out of which a regional core collection of 196 accessions was formed, and the CePaCT was mandated to conserve and share accessions of this core collection with Pacific Island countries for breeding purposes. With funds provided by ACIAR, the University of Queensland supported DNA fingerprinting and virus indexing of the taro core collection.^6^ This approach allowed the safe movement of germplasm from the CePaCT to the Taro Improvement Project (TIP) which was established with support from TaroGen at the University of the South Pacific in Samoa.^35^

Crop-focused participatory appraisals were conducted with farmers' groups to elicit taro production constraints and farmers' perceptions of key taro traits for the selection of taro cultivars.^36^ The CePaCT genebank supported the participatory breeding program in Samoa through the provision of more diverse taro germplasm imported from the Asian gene pool. With support from the European Commission INCO-DC program, the Taro Network for Southeast Asia and Oceania (TANSAO) was established in 1998 and administered by CIRAD (Centre de Coopération Internationale en Recherche Agronomique pour le Développement). Under this network, 2300 accessions and elite cultivars were collected from Southeast Asia and the Pacific, and a core set of 168 accessions has been established based on morphological and isozyme data, representative of the genetic diversity of the countries involved.^27^

The core set was transferred to the CePaCT genebank, virus tested, and selected accessions were made available by CePaCT for use by the national breeding programs in Samoa and Vanuatu. This approach allowed the breeding of new taro cultivars with a much broader genetic diversity for stable disease resistance, drought tolerance, and good palatability. Elite breeding lines from the TIP were shared with the CePaCT genebank for virus indexing, inclusion into the regional collection, and for sharing with other countries in the Pacific.^6^ As the CePaCT genebank collections were designated to the International Treaty in 2009, elite taro lines conserved by CePaCT are now available globally. Table 3 gives an overview of the global taro germplasm distribution by CePaCT from 2004 to 2017. During this period 3059 accessions and close to 22,000 plant samples were successfully distributed. The host country Fiji and PNG received the largest share of taro germplasm. Most distributions outside of the Pacific were funded by the International Network of Edible Aroids (INEA) project.

**Table 3.** Global Distribution of Taro Germplasm by the CePaCT During the Period of 2004–2017

*Recipient country*	*No. of accessions*	*No. plant samples*	*Recipient country*	*No. of accessions*	*No. plant samples*
American Samoa	128	728	Mauritius	10	50
Australia	16	148	Nauru	38	148
Bangladesh	12	60	New Caledonia	18	118
Belgium	8	130	Nicaragua	50	412
Burkina Faso	50	432	Nigeria	60	544
Cameroon	20	80	Niue	65	377
Comoros	4	20	Norfolk Island	22	74
Congo	15	75	Northern Marianas	22	74
Cook Islands	84	662	Palau	75	388
Costa Rica	50	376	Philippines	50	448
Cuba	50	416	Pitcairn Islands	28	146
Fiji	500	2915	Papua N. Guinea	619	4917
Federated States of Micronesia	62	411	Portugal	15	125
Germany	12	55	Samoa	65	799
Ghana	50	422	Solomon Islands	104	682
Guadeloupe	17	80	South Africa	50	450
Guam	22	108	Thailand	24	121
Haiti	8	62	Tokelau	28	180
India	50	420	Tonga	35	287
Indonesia	50	408	Trinidad and Tobago	50	495
Kenya	50	454	Tuvalu	27	155
Kiribati	109	660	Vanuatu	66	521
Madagascar	50	458	Wallis and Futuna	45	309
Marshall Islands	76	545			
Grand total	3059	21,945			

With the recent TLB outbreak and spread in West Africa, in Nigeria,^37^ the global leader in taro production, and Ghana,^38^ also among the top five global producers, access to TLB-resistant germplasm became vital. However, introducing new allelic diversity of taro globally is not only relevant for TLB resistance breeding but also for better adaptation to abiotic stresses exacerbated by climate change.

SPC-CePaCT, one of the lead organizations of the INEA project, funded by the European Union, responded to this global threat and provided in most cases 50 virus-indexed taro genotypes to 15 countries in Africa (Burkina Faso, Ghana, Kenya, Madagascar, Nigeria, and South Africa), Latin America (Costa Rica and Nicaragua), the Caribbean (Cuba, Trinidad, and Tobago), Asia (India, Indonesia, and Philippines), and the Pacific (PNG, Vanuatu) during the second half of 2011.

The performance of the genotypes distributed under the INEA project was evaluated on-station and compared to elite local germplasm.^39^ The breeding lines introduced from Hawaii, PNG, and Samoa outperformed local varieties in most countries in terms of yield and TLB tolerance. Surprisingly, several elite, TLB-tolerant cultivars from Southeast Asia performed better than the breeding lines from Hawaii, PNG, and Samoa in four countries.^39^ In South Africa, none of the 50 introduced genotypes performed better than local varieties. The best performers were distributed to farmers for participatory on-farm evaluation. In addition, introduced genotypes were successfully crossed with local cultivars to create novel diversity for future challenges.

## Conclusions

The genetic diversity of a clonally propagated crop like taro is expected to be narrow, especially in countries where it was introduced through vegetative propagules. This narrow genetic base not only makes the crop extremely susceptible to biotic stresses like TLB disease but also to abiotic stresses. Collecting and conserving the existing wild and cultivated genetic diversity in the countries of origin and diversity and sharing this diversity with producer countries in other parts of the world, in Africa, the Americas, and the Caribbean is a strongly recommended approach. The CePaCT genebank in Fiji has assumed this role of conservation of taro genetic resources, virus indexing, and safe sharing of this resource with taro germplasm users worldwide. Crossing and backcrossing introduced germplasm with local elite cultivars is an assurance for farmers that the newly created cultivars are more likely to withstand emerging biotic and abiotic stresses, exacerbated by climate change.

CePaCT is in the process of engaging with other national stakeholders to strengthen national seed/planting material systems and to ensure that farmers benefit from elite germplasm conserved at and shared by the Center. With an efficient droplet vitrification protocol in place that results in post-thaw regeneration rates between 70% and 100%, applicable to a wide range of taro genotypes, CePaCT is now planning to establish a cryopreservation facility for long-term preservation of taro and, thereafter, other major crops currently conserved in tissue culture at the Center. Eventually, this will also include the long-term cryopreservation of coconut genetic resources of the Pacific.

## References

[1] Matthews PJ, Lockhart PJ, Ahmed I Phylogeography, ethnobotany and linguistics issues arising from research on the natural and cultural history of taro, *Colocasia esculenta* (L.) Schott. Man India 2017;97:353–380

[2] Matthews PJ Genetic diversity in taro, and the preservation of culinary knowledge. Ethnobotany Res Appl 2004;2:55–71

[3] FAO (Food and Agriculture Organization of the United Nations). 2017 Available at www.fao.org/faostat/en/#data/QC; accessed on 410, 17

[4] Jianchu X, Yongping Y, Yingdong P, Ayad WG, Eyzaguirre PB Genetic diversity in taro *(Colocasia esculenta* Schott, Araceae) in China: An ethnobotanical and genetic approach. Econ Botany 2001;55:14–31

[5] Ramanatha Rao V, Matthews PJ, Eyzaguirre PB, Hunter D (Editors). The Global Diversity of Taro: Ethnobotany and Conservation. Rome, Italy: Bioversity International; 2010

[6] Iosefa TL, Taylor M, Hunter D, Tuia V The taro improvement programme in Samoa: Sharing genetic resources through networking. In: FAO RAP-NIAS. Plant Genetic Resources for Food and Agriculture in Asia and the Pacific: Impacts and future directions. Proceedings of a symposium held in Tsukuba, Japan Bangkok, Thailand: FAO Regional Office for Asia and the Pacific; 2012: 25–40

[7] Chaïr H, Traore RE, Duval MF, et al. Early and mid Holocene tool-use and processing of taro (*Colocasia esculenta*), yam (*Dioscorea* sp.) and other plants at Kuk Swamp in the highlands of Papua New Guinea. J Archaeol Sci 2006;33:595–614

[8] Loy TH, Spriggs M, Wickler S Direct evidence for human use of plants 28,000 years ago: Starch residues on stone artefacts from the northern Solomon Islands. Antiquity 1992;66:898–912

[9] Fullagar R, Field J, Denham T, Lentfer C Early and mid Holocene tool-use and processing of taro (*Colocasia esculenta*), yam (*Dioscorea* sp.) and other plants at Kuk Swamp in the highlands of Papua New Guinea. J Archaeol Sci 2006;33:595–614

[10] Horrocks M, Nunn PD Evidence for introduced taro (*Colocasia esculenta*) and lesser yam (*Dioscorea esculenta*) in Lapita-era (c. 3050–2500 cal. yr BP) deposits from Bourewa, southwest Viti Levu Island, Fiji. J Archaeol Sci 2007;34:739–748

[11] Lebot V Tropical root and tuber crops: Cassava, sweet potato, yams and aroids. CABI 2009;17:413

[12] Beaujard P The first migrants to Madagascar and their introduction of plants: Linguistic and ethnological evidence. Azania Archaeol Res Afr 2011;46:169–189

[13] Blench R Bananas and plantains in Africa: Re-interpreting the linguistic evidence. Ethnobotany Res Appl 2009;7:363–380

[14] Manzano AR, Nodals AA, Gutiérrez MI, Mayor ZF, Alfonso LC Morphological and isoenzyme variability of taro (*Colocasia esculenta* L. Schott) germplasm in Cuba. Plant Genet Resour Newsl 2001;126:31–40

[15] Gonçalves RF, Silva AM, Silva AM, et al. Influence of taro (*Colocasia esculenta* L. Schott) growth conditions on the phenolic composition and biological properties. Food Chem 2013;141:3480–3485 23993510 10.1016/j.foodchem.2013.06.009

[16] Mathews P, Matsushita Y, Sato T, Hirai M Ribosomal and mitochondrial DNA variation in Japanese taro *(Colocasia esculenta* L. Schott). Jpn J Breed 1992;42:825–833

[17] Tahara M, Suefuji S, Ochiai T, Yoshino H Phylogenetic relationships of taro, *Colocasia esculenta* (L.) Schott and related taxa by non-coding chloroplast DNA sequence analysis. Aroideana 1999;22:79–89

[18] Irwin SV, Kaufusi P, Banks K, De La Peña R, Cho JJ Molecular characterization of taro (*Colocasia esculenta*) using RAPD markers. Euphytica 1998;99:183–189

[19] Lebot V, Aradhya KM Isozyme variation in taro (*Colocasia esculenta* (L.) Schott) from Asia and Oceania. Euphytica 1991;56:55–66

[20] Zhang D, Zhang G Preliminary studies on evolution and classification of taro (*Colocasia* spp.) in China. In: Zhu D, Eyzaguirre PB, Zhou M, Sears L and Liu G (eds). Ethnobotany and genetic diversity of Asian taro: Focus on China. Proceedings of the Symposium on Ethnobotanical and Genetic Study of Taro in China: Approaches for the Conservation and Use of Taro Genetic Resources; 10–12 November 1998—Laiyang Agricultural College, Laiyang, Shandong, China. Beijing: IPGRI Office for East Asia; 2000: 32–45

[21] Lebot V, Prana MS, Kreike N, et al. Characterisation of taro (*Colocasia esculenta* (L.) Schott) genetic resources in Southeast Asia and Oceania. Genet Resour Crop Evol 2004;51:381–392

[22] Kreike CM, Van Eck HJ, Lebot V Genetic diversity of taro, *Colocasia esculenta* (L.) Schott, in Southeast Asia and the Pacific. Theor Appl Genet 2004;109:761–768 15156282 10.1007/s00122-004-1691-z

[23] Lebot V Biomolecular evidence for plant domestication in Sahul. Genet Resour Crop Evol 1999;46:619–628

[24] Ochiai T, Nguyen VX, Tahara M, Yoshino H Geographical differentiation of Asian taro, *Colacasia esculenta* (L.) Schott, detected by RAPD and isozyme analyses. Euphytica 2001;122:219–234

[25] FAO (Food and Agriculture Organization of the United Nations). World Information and Early Warning System (WIEWS) on Plan Genetic Resources for Food and Agriculture. Ex Situ search. 2018 Available at www.fao.org/wiews/data/ex-situ-sdg-251/search/en/; accessed on 612, 18

[26] Genesys. Statistical overview. 2018 Available at www.genesys-pgr.org/explore/overview?filter=%7B%22crops%22%3A%5B%22taro%22%5D%7D; accessed on 128, 18

[27] Anonymous. Edible aroids conservation strategy. 2010 Available at: www.genebanks.org/wp-content/uploads/2017/01/Edible-Aroids-Strategy-2010.pdf

[28] Mace ES, Mathur PN, Izquierdo L, et al. Rationalization of taro germplasm collections in the Pacific Island region using simple sequence repeat (SSR) markers. Plant Genet Resour 2006;4:210–220

[29] Singh D, Mace E, Okpul T, et al. Collection, characterization and conservation of taro (*Colocasia esculenta*) genetic resources for efficient utilization in breeding. In 12th Australasian Plant Breeding Conference 2002 (Vol. 1, pp. 251–253). The Australasian Plant Breeding Association, Inc

[30] Dulloo ME, Ebert AW, Dussert S, et al. Cost efficiency of cryopreservation as a long-term conservation method for coffee genetic resources. Crop Sci 2009;49:2123–2138

[31] Taylor M, Hunter D, Rao VR, Jackson GV, Sivan P, Guarino L Taro collecting and conservation in the Pacific region. In: Ramanatha Rao V, Matthews PJ, Eyzaguirre PB, Hunter D (eds). The Global Diversity of Taro: Ethnobotany and Conservation. Rome, Italy: Bioversity International; 2010: 150–167

[32] Bessembinder JJ, Staritsky G, Zandvoort EA Long-term in vitro storage of *Colocasia esculenta* under minimal growth conditions. Plant Cell Tissue Organ Cult 1993;33:121–127

[33] Sant R, Panis B, Taylor M, Tyagi A Cryopreservation of shoot-tips by droplet vitrification applicable to all taro (*Colocasia esculenta* var. *esculenta*) accessions. Plant Cell Tissue Organ Cult 2008;92:107–111

[34] Misra RS, Sharma K, Mishra AK Phytophthora leaf blight of Taro (*Colocasia esculenta*)—a review. Asian Australas J Plant Sci Biotechnol 2008;2:55–63

[35] Hunter DG, Iosefa T, Delp CJ, Fonoti P Beyond taro leaf blight: A participatory approach for plant breeding and selection for taro improvement in Samoa. In: Proceedings of the International Symposium on Participatory Plant Breeding and Participatory Plant Genetic Resource Enhancement, CGIAR System-Wide Program on Participatory Research and Gender Analysis for Technology Development and Institutional Innovation, Cali, Colombia, CIAT; 2001: 219–227

[36] Singh D, Hunter D, Iosefa T, Okpul T, Fonoti P, Delp C Improving taro production in the South Pacific through breeding and selection. In: Ramanatha Rao V, Matthews PJ, Eyzaguirre PB, Hunter D (eds). The Global Diversity of Taro: Ethnobotany and Conservation. Rome, Italy: Bioversity International; 2010: 168–184

[37] Bandyopadhyay R, Sharma K, Onyeka TJ, Aregbesola A, Kumar PL First Report of Taro (*Colocasia esculenta*) Leaf Blight Caused by *Phytophthora colocasiae* in Nigeria. Plant Dis 2011;95:618 10.1094/PDIS-12-10-0890 30731969

[38] Omane E, Oduro KA, Cornelius EW, et al. First report of leaf blight of taro (*Colocasia esculenta*) caused by *Phytophthora colocasiae* in Ghana. Plant Dis 2012;96:292 10.1094/PDIS-09-11-0789 30731838

[39] Lebot V, Tuia V, Ivancic A, et al. Adapting clonally propagated crops to climatic changes: A global approach for taro (*Colocasia esculenta* (L.) Schott). Genet Resour Crop Evol 2018;65:591–606

